# Supplementary figures and images for: Transcriptomic basis and evolution of the ant nurse-larval social interactome
Source: PLoS Genet. 2019 May 20;15(5):e1008156. doi: 10.1371/journal.pgen.1008156 (PMC6544314; doi:10.1371/journal.pgen.1008156)

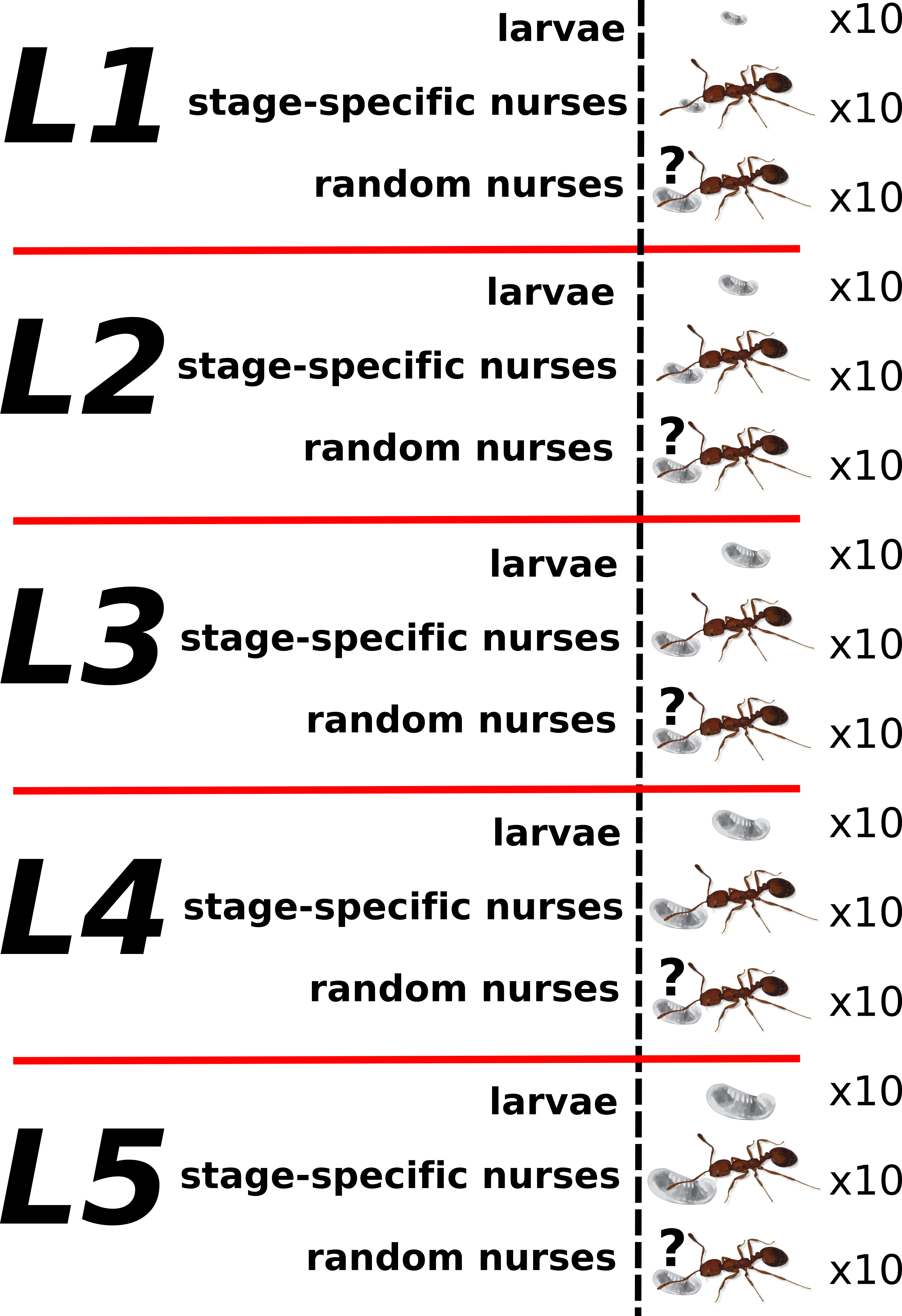

Supplement: S1 Fig — We collected ten worker-destined larvae, ten stage-specific nurses, and ten random nurses from each colony (six colonies per time point, where time points represent larval developmental stages L1, L2, etc). We collected stage-specific nurses when we observed them feeding larvae of the given developmental stage. We collected random nurses when we observed them feeding any stage of larvae. (TIF) [file pgen.1008156.s001.tif]

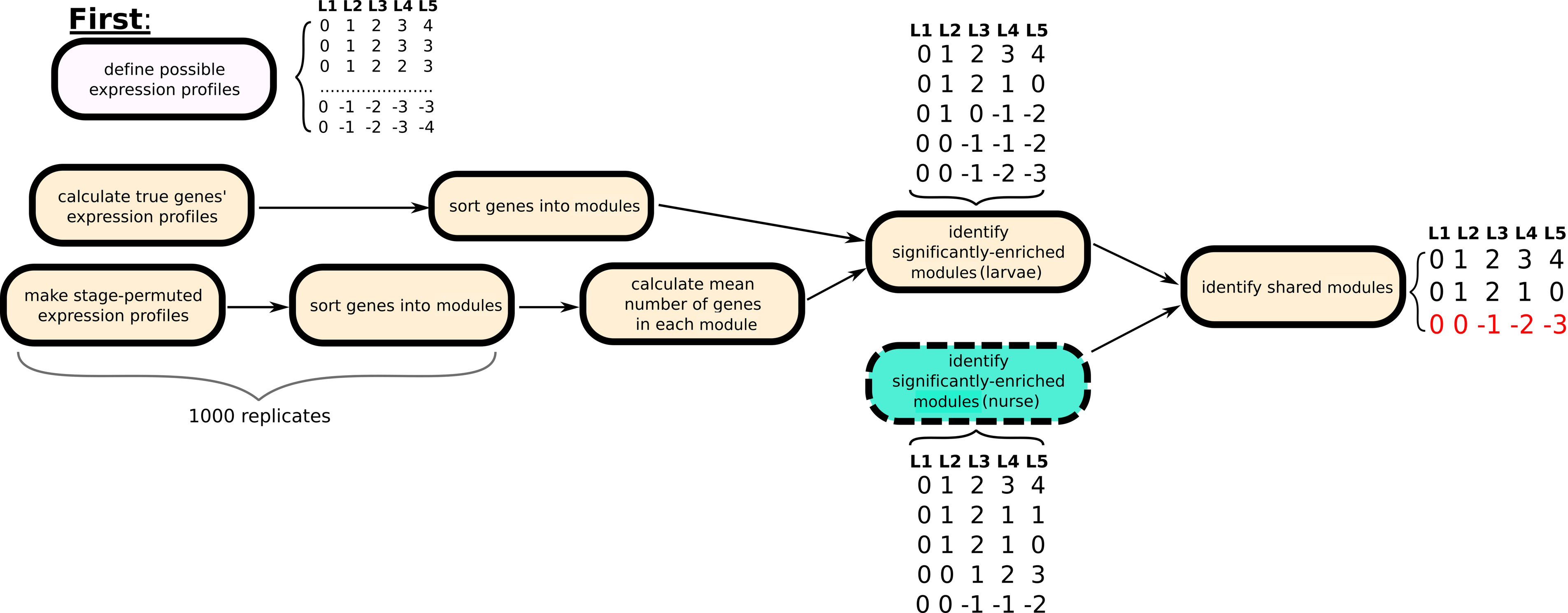

Supplement: S2 Fig — Inset tables depict pre-defined expression profiles of modules genes can be assigned to. First, we construct modules using all possible expression profiles (top left bubble). Expression profiles consist of five values, starting at zero, that indicate the log2 fold-change in expression from the initial value (at stage L1). At each subsequent stage, we either double, halve, or keep the expression level the same. This process is repeated to produce 81 (four stages after L1; 3*3*3*3 = 81) total modules. Next, for each tissue separately (here we depict workflow in larvae with yellow bubbles), we calculate individual gene expression profiles as the log2 fold-change in expression from the initial value at stage L1 and assign genes to the closest related module by Pearson correlation. Concurrently, we permute the developmental stage labels for each gene and assign the stage-permuted genes to modules (repeated 1000 times). From these stage-permuted results, we calculate the mean number of genes assigned to each module and treat this number as a null expectation (as each expression profile is not equally likely to occur by chance). We then identify significantly-enriched modules using a one-way binomial test (with the calculated mean as the null), with a Bonferroni-corrected false discovery rate of 0.05. This entire process is repeated in a nurse tissue and significantly-enriched modules are found (blue bubble). Finally, we compare significantly-enriched modules between larvae and nurses and retain identical and inverse modules as shared profiles. An example of an inversely related profile is shown in red, where larvae exhibit the enriched module [0, 0, –1, –2, –3] and nurses exhibit the inverse module, [0, 0, 1, 2, 3]. (TIF) [file pgen.1008156.s002.tif]

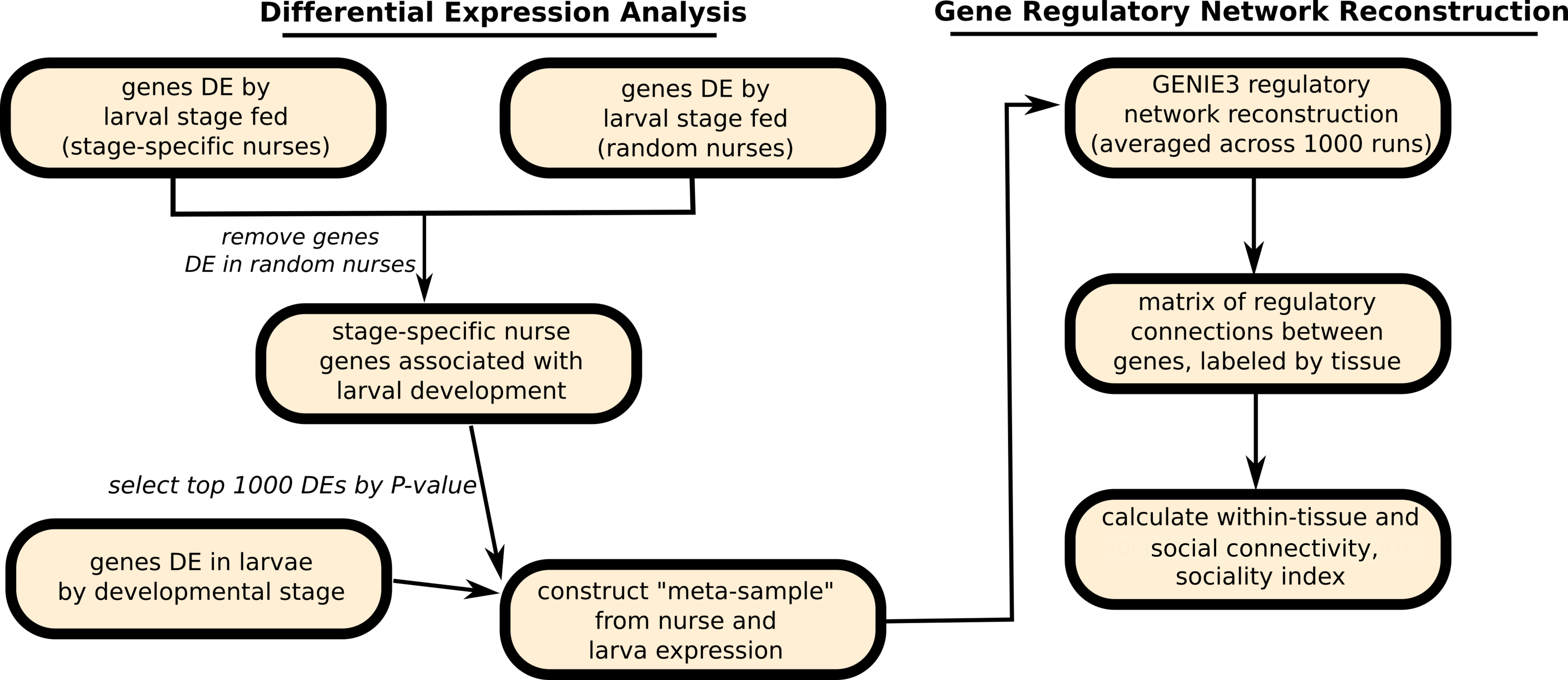

Supplement: S3 Fig — On the left, we identify putatively socially-acting genes through differential expression analysis. First, for nurse heads and abdomens separately, we perform differential expression analysis in stage-specific and random nurses to identify genes differentially expressed according to larval stage fed, using a nominal P-value of 0.05. We remove genes differentially expressed in random nurses, as these correspond to colony-specific environmental effects unrelated to social regulation of larval development. Next, we select the top 1000 differentially expressed genes by P-value in stage-specific nurses (after removing those DE in random nurses) as well as the top 1000 differentially expressed genes in larvae. From these genes, we create “meta-samples” by combining gene expression of larvae and stage-specific nurses collected from the same colony (separately for heads and abdomens), and labeling genes by the tissue they are expressed in. Using these meta-samples, we perform gene regulatory reconstruction (right) to identify genes expressed in nurses that regulate larval gene expression, and vise-versa. We repeat gene regulatory reconstruction 1000 times and average connection strength across runs, as the algorithm is non-deterministic. The output of gene regulatory reconstruction is a matrix of regulatory connections acting between genes. From this matrix, we calculate the average connectivity for each gene, separating within-tissue (larva-larva or nurse head-nurse head) from social (nurse-larva) connections. Genes with high connectivity are predicted to interact with many genes, i.e. are central to the network. Finally, we calculate each genes’ sociality index as the difference between social connectivity and within-tissue connectivity. (TIF) [file pgen.1008156.s003.tif]

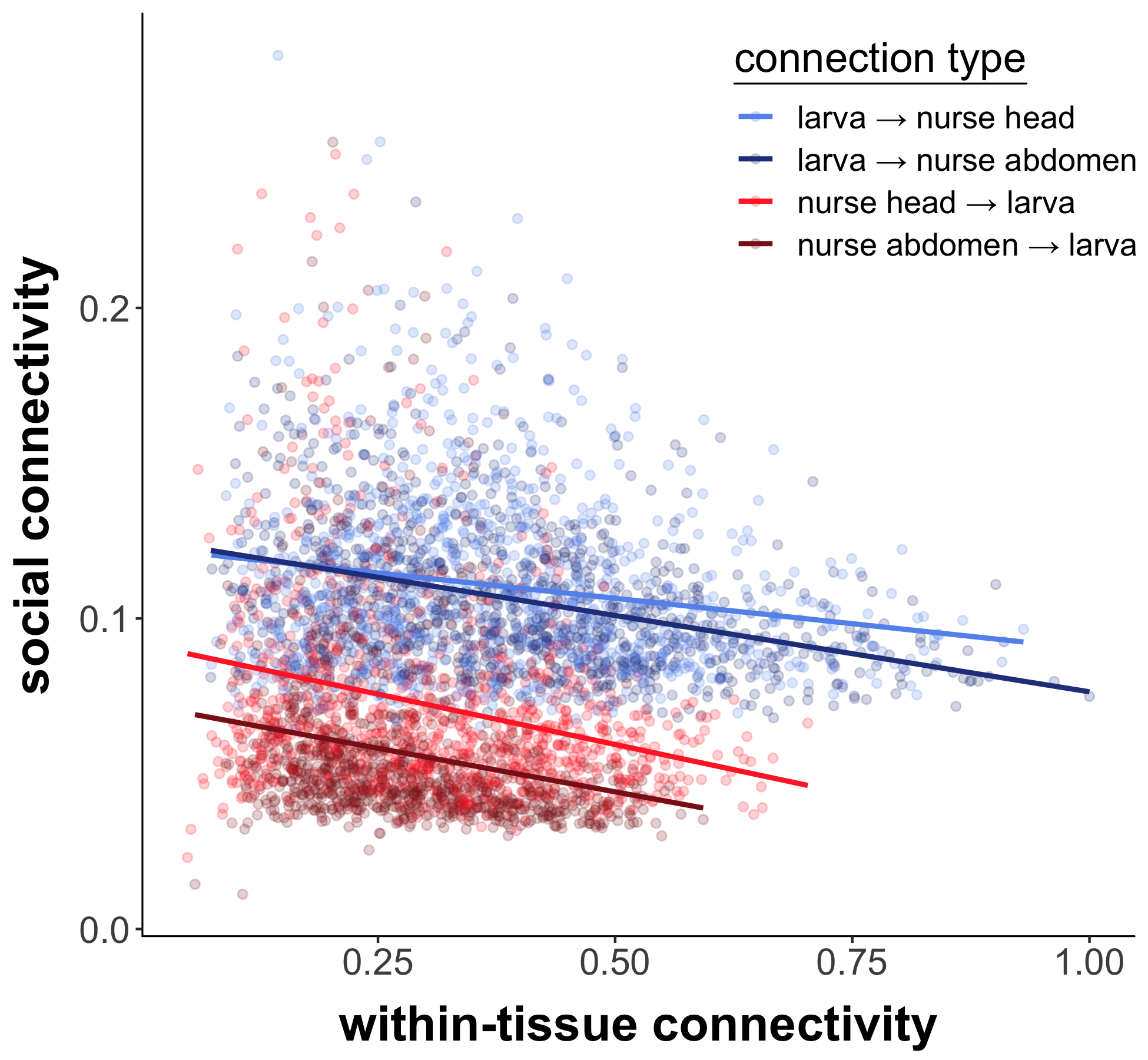

Supplement: S4 Fig — Connectivity is representative of the number and strength of regulatory connections each gene makes. Points indicate the average connectivity for a given gene, as measured within-tissue (x-axis; i.e. larva-larva or nurse-nurse) or socially (y-axis; i.e. larva-nurse). Points are colored by tissue the connectivity is measured in (e.g., dark blue indicates genes expressed in larvae, with connectivity measured in networks constructed with nurse abdomens). Spearman rho = -0.166, -0.374, -0.276, -0.342 for the four tissues as ordered in legend; P < 0.001 in all cases. (TIF) [file pgen.1008156.s004.tif]

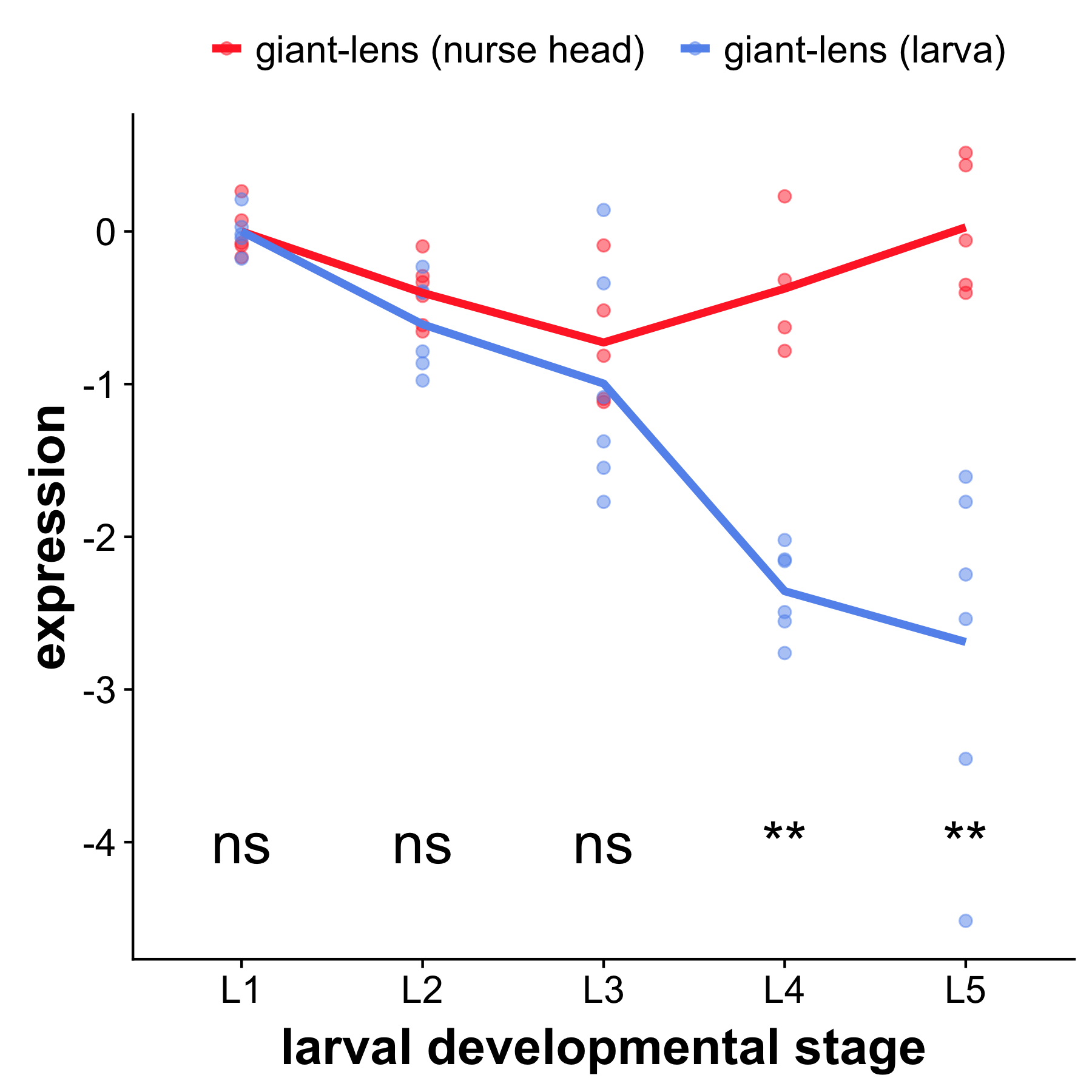

Supplement: S5 Fig — Expression at stage i is equal to log2(expressioni/expression1), i.e. the ratio of expression at the given stage to expression at the initial (L1) stage. **: P < 0.01, ns: P > 0.05 (Wilcoxon test at each stage). (TIF) [file pgen.1008156.s005.tif]

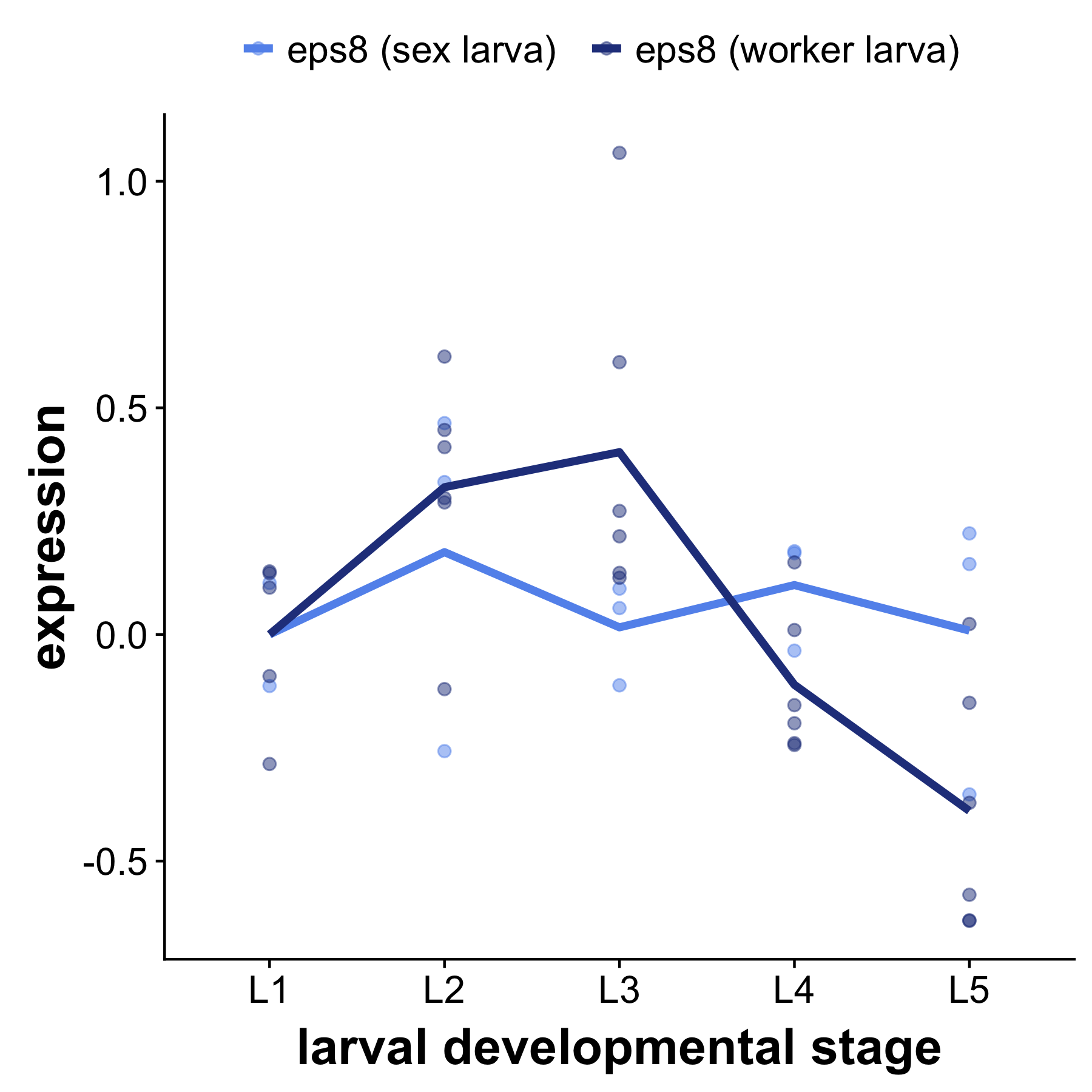

Supplement: S6 Fig — Expression at stage i is equal to log2(expressioni/expression1), i.e. the ratio of expression at the given stage to expression at the initial (L1) stage. Expression of eps8 changed differently over time in worker-destined versus reproductive-destined larvae (linear model with developmental stage treated as an ordinal variable; LRT; χ2 = 12.574, P = 0.014 for the interaction term stage*caste). (TIF) [file pgen.1008156.s006.tif]

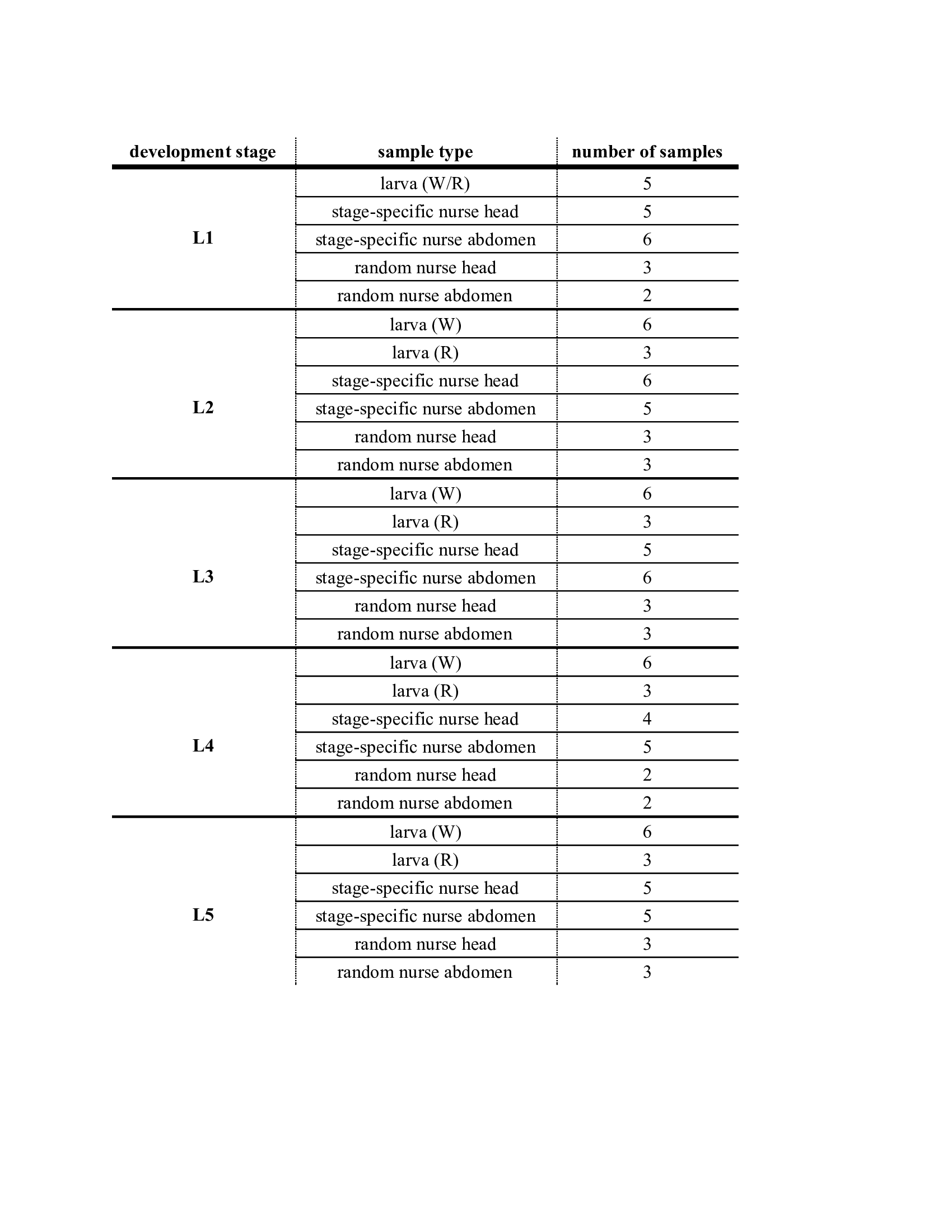

Supplement: S1 Table — Worker-destined larvae are indicated by larva (W), and reproductive-destined larvae are indicated by larva (R). Larval caste cannot be distinguished at the L1 stage, so L1 larvae are labeled larva (W/R). For network reconstruction, “meta” samples were used as input for network reconstruction, in which genes were labeled by sample type and grouped such that each gene contained a measurement of expression in worker-destined larvae, nurse heads, and nurse abdomens. After sample collection and RNA extraction, some samples exhibited clearly degraded RNA according to an Agilent Bioanalyzer assay. Removing these samples caused sampling to be uneven, so we used the minimum number of samples contained across tissues at a given stage for stage-specific nurse heads and abdomens, and randomly dropped excess samples. Overall, 25 “aggregate” samples were used as input for gene regulatory network reconstruction. (TIF) [file pgen.1008156.s007.tif]

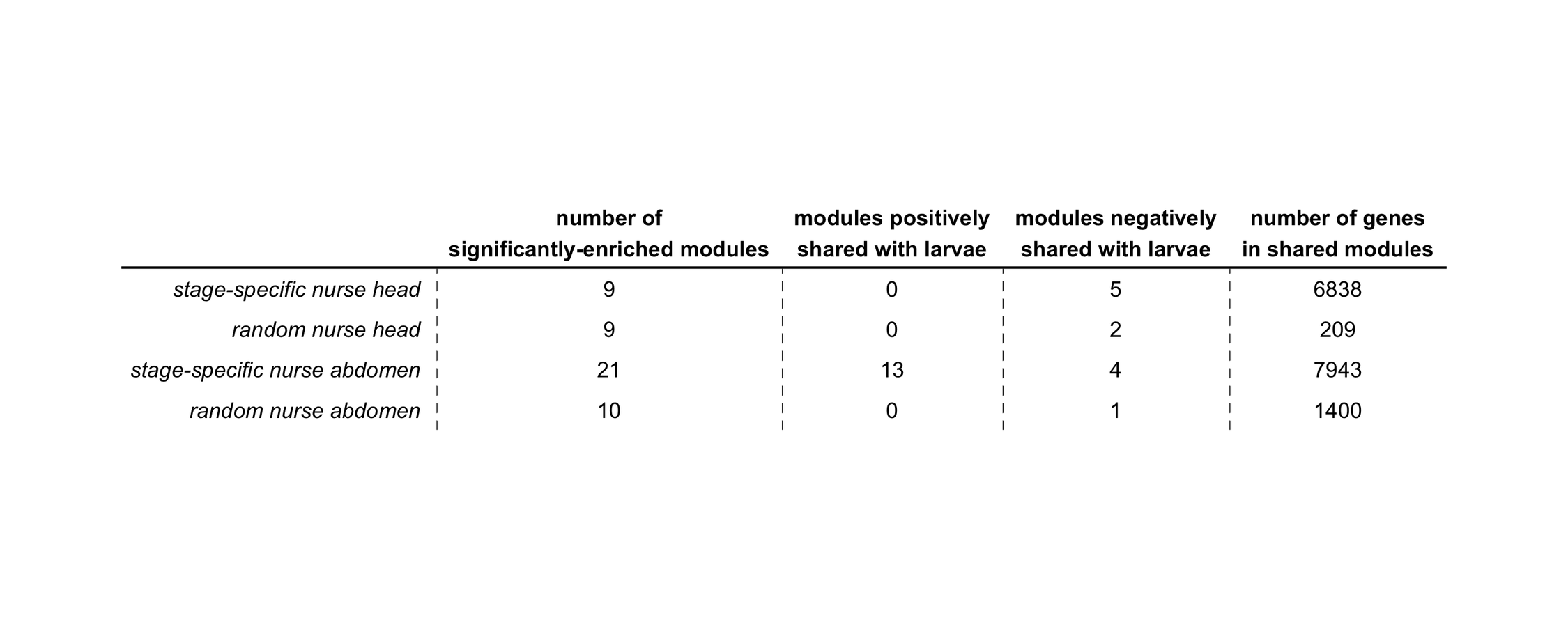

Supplement: S2 Table — Significantly-enriched modules are defined as modules with a statistically significant number of genes assigned, as determined by a permutation test (FDR < 0.05). Left column is the total number of significant modules for each tissue, while the second and third columns indicate number shared with larvae (out of 24 larval significantly-enriched modules). The last column indicates the total number of genes in these shared modules. (TIF) [file pgen.1008156.s008.tif]

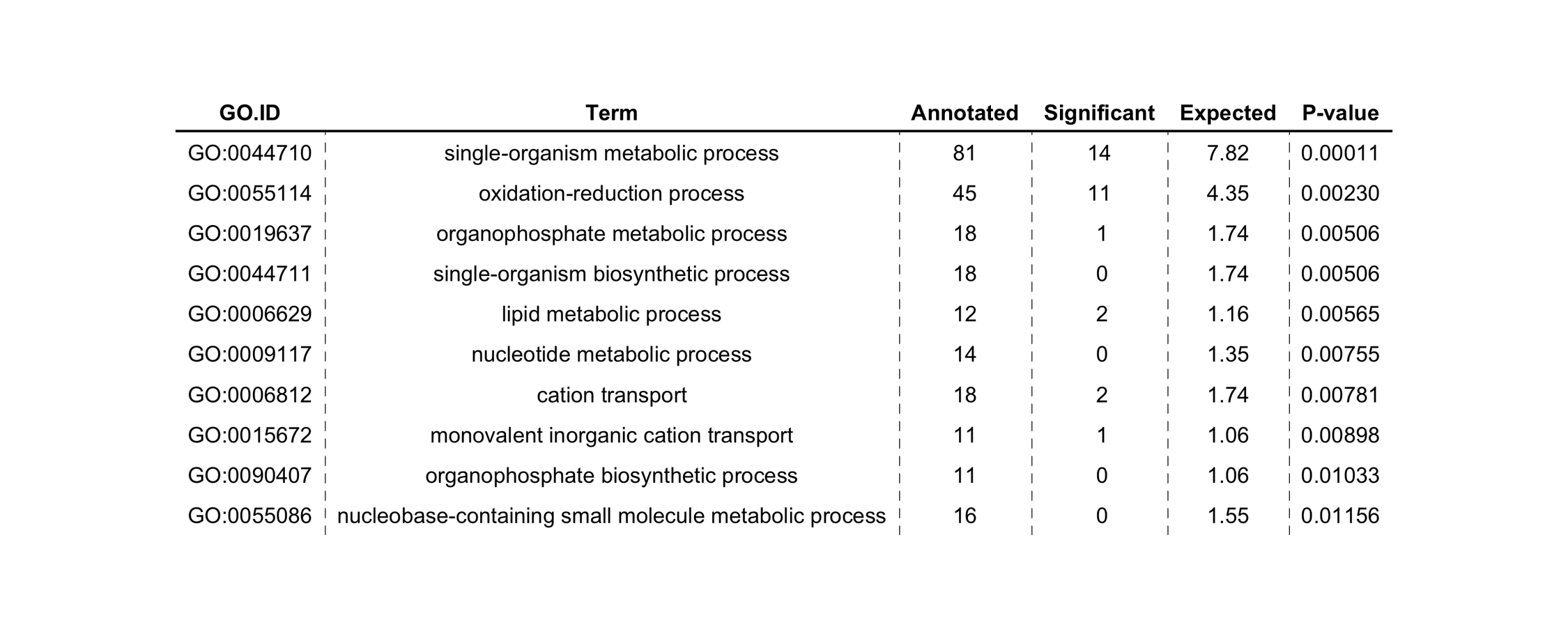

Supplement: S3 Table — P-value (unadjusted) is from Kolmogorov Smirnov (K-S) test. Enriched terms have higher than expected social connectivity in nurse heads. (TIF) [file pgen.1008156.s009.tif]

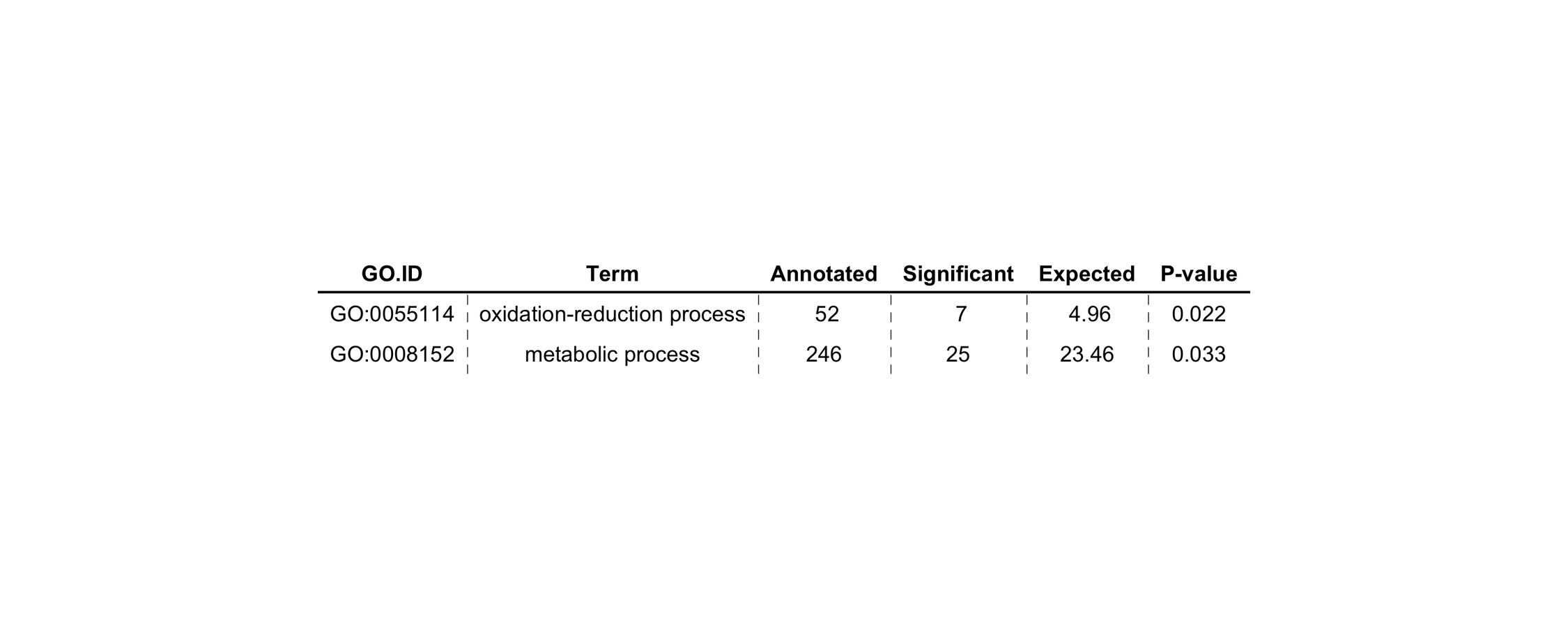

Supplement: S4 Table — P-value (unadjusted) is from Kolmogorov Smirnov (K-S) test. Enriched terms have higher than expected social connectivity in nurse abdomens. (TIF) [file pgen.1008156.s010.tif]

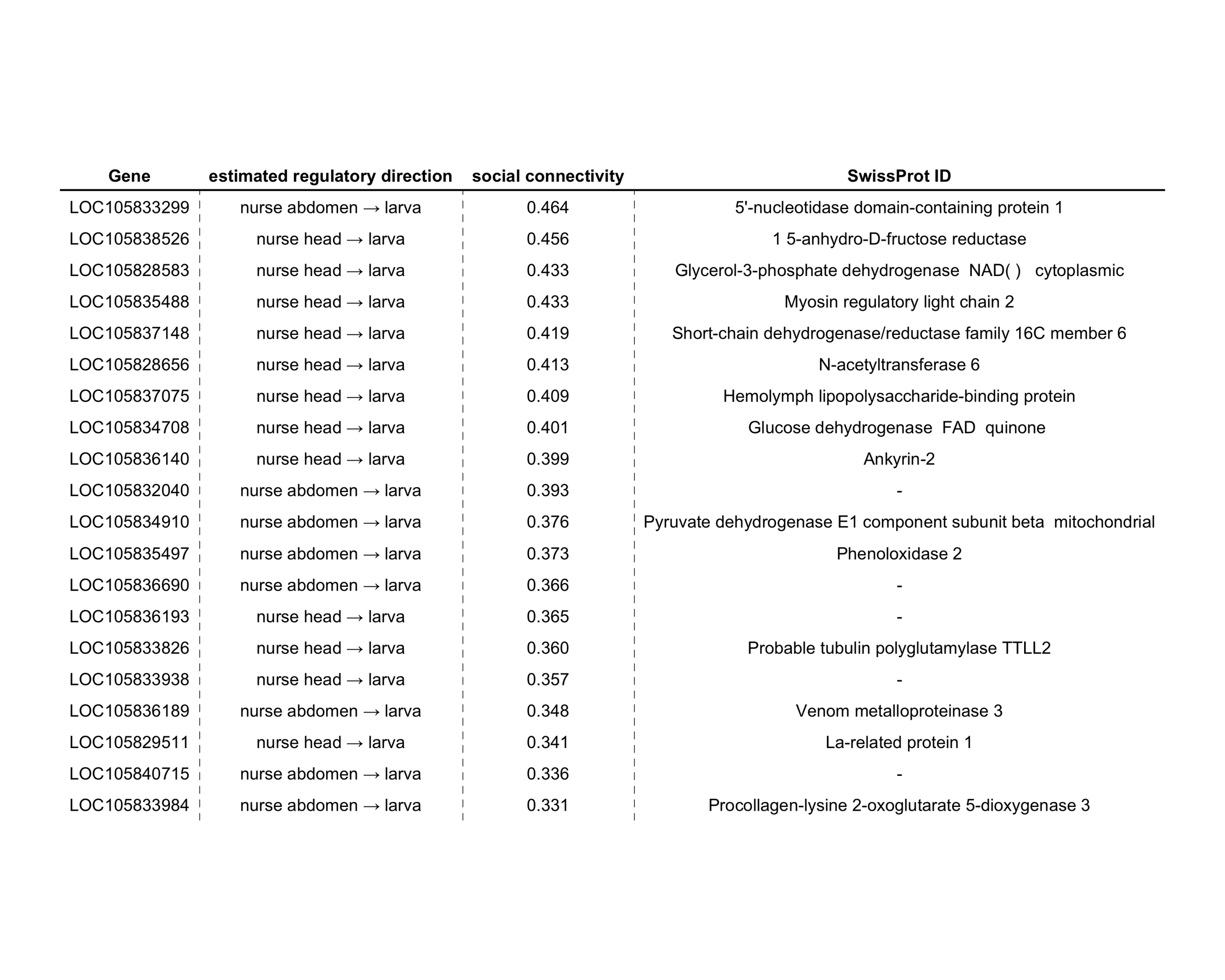

Supplement: S5 Table — SwissProt ID is listed from automated annotation where a term was found. (TIF) [file pgen.1008156.s011.tif]

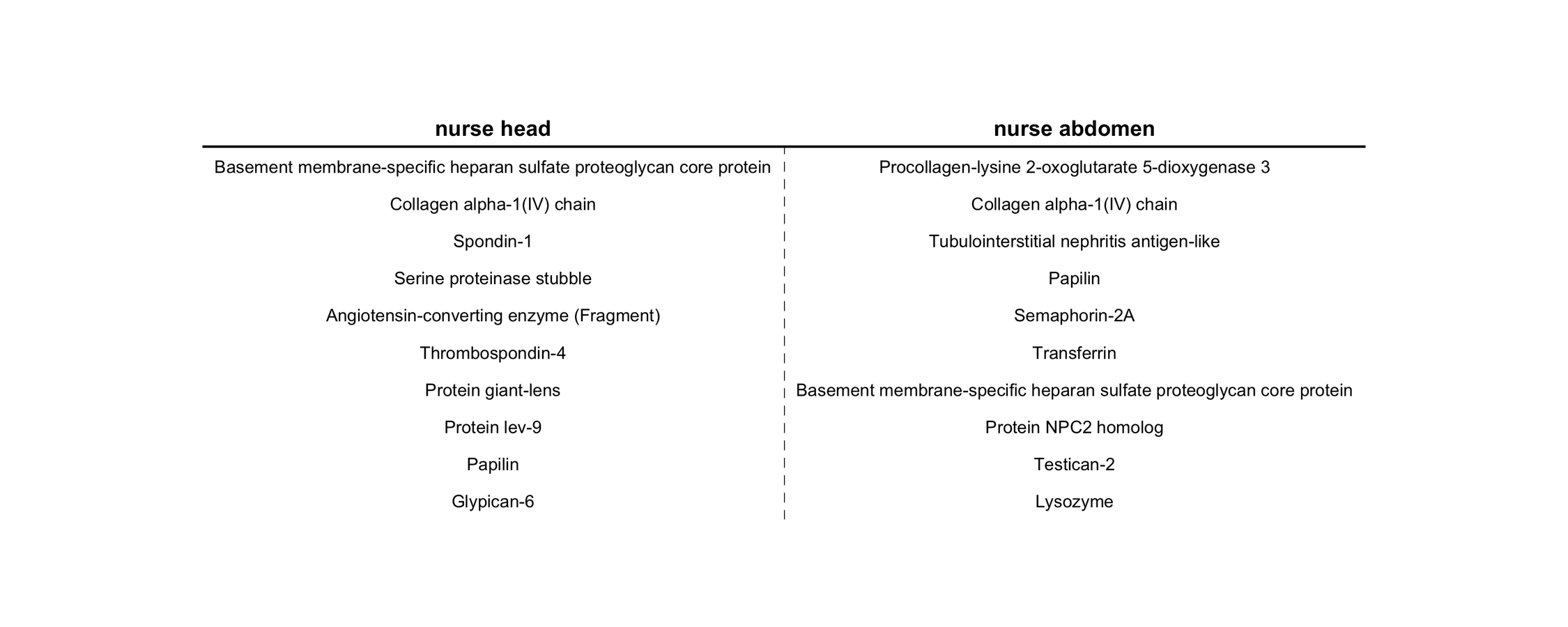

Supplement: S6 Table — Only genes with SwissProt annotations are included. All genes listed encode for secreted proteins in D. melanogaster. (TIF) [file pgen.1008156.s012.tif]
